# Phenotypic Characterization of Toxic Compound Effects on Liver Spheroids Derived from iPSC Using Confocal Imaging and Three-Dimensional Image Analysis

**DOI:** 10.1089/adt.2016.729

**Published:** 2016-09-01

**Authors:** Oksana Sirenko, Michael K. Hancock, Jayne Hesley, Dihui Hong, Avrum Cohen, Jason Gentry, Coby B. Carlson, David A. Mann

**Affiliations:** ^1^ Molecular Devices, LLC, Sunnyvale, California.; ^2^ Cellular Dynamics International—A Fujifilm Company, Madison, Wisconsin.

## Abstract

*Cell models are becoming more complex to better mimic the* in vivo *environment and provide greater predictivity for compound efficacy and toxicity. There is an increasing interest in exploring the use of three-dimensional (3D) spheroids for modeling developmental and tissue biology with the goal of accelerating translational research in these areas. Accordingly, the development of high-throughput quantitative assays using 3D cultures is an active area of investigation. In this study, we have developed and optimized methods for the formation of 3D liver spheroids derived from human iPS cells and used those for toxicity assessment. We used confocal imaging and 3D image analysis to characterize cellular information from a 3D matrix to enable a multi-parametric comparison of different spheroid phenotypes. The assay enables characterization of compound toxicities by spheroid size (volume) and shape, cell number and spatial distribution, nuclear characterization, number and distribution of cells expressing viability, apoptosis, mitochondrial potential, and viability marker intensities. In addition, changes in the content of live, dead, and apoptotic cells as a consequence of compound exposure were characterized. We tested 48 compounds and compared induced pluripotent stem cell (iPSC)-derived hepatocytes and HepG2 cells in both two-dimensional (2D) and 3D cultures. We observed significant differences in the pharmacological effects of compounds across the two cell types and between the different culture conditions. Our results indicate that a phenotypic assay using 3D model systems formed with human iPSC-derived hepatocytes is suitable for high-throughput screening and can be used for hepatotoxicity assessment* in vitro.

## Introduction

Liver injury caused by toxicity of pharmaceutical drugs and environmental chemicals is a subject of great concern. Therefore, development of new complex systems that would allow effective testing for potential liver toxicity is an area of active investigation.^1–3^ There is a growing interest in using three-dimensional (3D) models for studying complex biology and tissue engineering. Recently, 3D models have been employed for developmental biology research and toxicity screening.^4–9^

Numerous studies have reported more liver-specific functional activity in hepatocyte 3D cultures than in conventional two-dimensional (2D) monolayers,^5,10–12^ since mechanical stress mediated by adhesion to extracellular matrix or other cells alters signal transduction and gene transcription in a variety of cell types. In the human liver hepatocellular carcinoma cell line (HepG2), cells respond to differing physical environments of 2D and 3D culture with altered actin cytoskeleton structure and cell shape. Further, global gene expression analysis has shown that distinct genetic programs are initiated depending on the physical structure of the cells: metabolic and synthetic functional genes, including cytochrome P450 and albumin, are upregulated in 3D spheroid structures.^1,5,7,10,13–22^

The majority of published liver toxicity studies have used transformed hepatocyte cell lines (HepG2, HepaRG) or primary hepatocytes for toxicity screening,^10,12,16,18,19,23^ whereas induced pluripotent stem cell (iPSC)-derived hepatocytes present a valuable model that can closely resemble the phenotypes and functionality of primary hepatocytes^11,13,22,24–29^ while minimizing variability and other limitations of primary cells. Human iPSC-derived hepatocytes show great promise with respect to having a primary tissue-like phenotype, consistent and unlimited availability, and the potential to establish genotype-specific cells from different individuals.

There has been significant progress in the development of 3D cell models and techniques during the past several years. Development strategies have included biodegradable scaffolds, organ-on-a chip structures, and self-assembled organoids.^24,29–31^ Recently, spheroid formation in low-attachment round-bottom plates has become popular, as the method offers a simple workflow and is compatible with high-content imaging.^7,17,23,32–35^ Common methods of analysis include disruption of spheroids and analysis of cell lysates or suspensions for ATP or other metabolites with microplate readers,^11^ whereas high-content imaging methods have been proven productive for the characterization of phenotypic effects of chemical compounds on morphology and viability.^33,36,37^

High-content imaging can be used with numerous fluorophores in combination, including stains for viability, DNA binding, apoptosis markers, or mitochondria markers.^19,38^ This method can be extended to more complex multicellular models that express a plurality of fluorescent markers. The use of higher magnification, as well as confocal imaging and 3D analysis provides single-cell resolution and characterization of cell content and morphology in 3D volume. Higher magnification confocal imaging and 3D analysis also allow derivation of multi-parametric outputs for characterizing complex phenotypes of spheroids treated with compounds.^33^

The goal of this study was to develop and characterize confocal high-content imaging in combination with 3D image analysis methods that would be suitable for the high-throughput compound screening using liver spheroids made from iPSC-derived hepatocytes. Sample handing steps for cell culture, treatment, and staining have been reduced to minimize spheroid disturbances and increase assay reproducibility. We optimized and compared imaging and image analysis methods and described measurements for multi-parametric characterization of different spheroid phenotypes and determination of IC_50_ values. Furthermore, we characterized the assay using 50 benchmark cytotoxic and known hepatotoxic compounds and compared IC_50_ values for 3D and 2D cultures. Finally, we compared this model with spheroids formed with HepG2, and we discovered significant differences in toxicity assessment between those systems. The method described here can enhance development of relevant cell-based assays for efficient assessment of the hepatotoxicity of chemicals and drug candidates in high-throughput quantitative screening.

## Materials and Methods

### Cell Models

Human iPSC-derived hepatocytes, iCell Hepatocytes 2.0 (Cellular Dynamics International), and HepG2 (ATCC) were used in the study. Cryopreserved cells were thawed and maintained according to provided protocols. To prepare spheroid cultures of human iPSC-derived hepatocytes, iCell Hepatocytes 2.0 were first thawed and plated at a high density (∼300,000 cells/cm^2^) onto collagen I-coated 24-well plates to allow the cells to recover from cryopreservation and to establish a confluent 2D culture.

After 7 days in culture, cells were gently detached using StemPro Accutase (ThermoFisher Scientific), pelleted by centrifugation, and resuspended in William's E Medium containing Hepatocyte Maintenance Supplement (ThermoFisher Scientific). Immediately before plating the cells, the cell suspension was further supplemented with a 10% final volume/volume of ready-to-use hESC-qualified Geltrex reduced growth factor basement membrane matrix (ThermoFisher Scientific). The combined cells plus matrix suspension was plated directly into 96-well GravityTRAP ultra-low attachment spheroid plates (InSphero) at ∼1,000 cells/well, which were immediately centrifuged (300 *g*, 2 min) to settle the cells and to remove any bubbles before placing the plate in a humidified incubator (37°C, 5% CO_2_). Spheroid formation was observed within 24–48 h.

HepG2 cells were cultured in minimum essential medium (Corning) supplemented with 10% fetal bovine serum and plated into 384-well ultra-low attachment (ULA) Corning U-shaped black clear-bottom plates (Corning 3830), at densities of 1,000 cells/well in the appropriate media.

### Chemicals and Treatments

For compound screening, test agents were prepared as 10–100 mM stock solutions in tissue culture-grade dimethyl sulfoxide (DMSO; Sigma-Aldrich); the final concentration of DMSO in media was 0.1%–0.3%. Compounds (Sigma-Aldrich) were tested in triplicate or duplicate, in a six-point dilution series. For spheroid assays, iCell Hepatocyte and HepG2 spheroids were plated 48 h before initiation of the experiment. Cells were then exposed to the indicated concentrations of compounds for 72 h. For mitochondria toxicity or caspase activation assay, spheroids were cultured for 48 h and then treated with compounds for 24 h. For conventional 2D assays, cells were plated on collagen-coated 384-well plates (BD Biocoat) for 48 h and then treated with compounds for 72 h (*Supplementary Fig. S1*).

### Multiparametric Live Cell Toxicity Assay

After incubation with test compounds, spheroids were stained with a mixture of three dyes: 2 μM calcein AM, 3 μM of EthD-1, and 10 μM Hoechst 33342 (Life Technologies). Dyes were prepared in sterile phosphate-buffered saline (Corning). In separate experiments, CellEvent Caspase 3/7 reagent (Life Technologies) was used to evaluate compound ability to trigger apoptosis signaling. Then, 7.5 μM CellEvent was added with 10 μM Hoechst nuclear dye diluted in Hank's Balanced Salt Solution (Corning). For mitochondria toxicity assay, MitoTracker Orange from Life Technologies (200 nM) was combined with Hoechst (10 μM). Dye solutions were added directly to the media without aspiration. Spheroids were incubated with dye for 2 h before imaging. Dye solution was not washed out, and care was taken during pipetting to avoid spheroid loss, disintegration, or displacement.

### Image Acquisition

Images were acquired using an ImageXpress Micro^®^ Confocal High-Content Imaging System (Molecular Devices), with a 20× Plan Fluor or 10× Plan Fluor objective. Typical image acquisition settings are given in *Supplementary Table S1* (Supplementary Data are available online at www.liebertpub.com/adt). A stack of 11–17 planes separated by 5–10 μm was acquired, starting at the well bottom and covering approximately the lower half of each spheroid. Typically, a Z-stack of images covered 100–120 μm for iCell Hepatocyte or HepG2 spheroids. All individual images were saved and used for 3D analysis, as well as for 2D projection (Maximum Projection [MaxPro]) images.

### 3D Image Analysis

Images were analyzed using the 3D analysis module of MetaXpress^®^ High-Content Image and Analysis Software (Molecular Devices). The analysis method is described in the Supplementary Data. Find Spherical Objects function was used to define spheroids. Then, Count Nuclei, Cell Scoring, and Live-Dead application modules were used for cell count and viability assessment.

A customized analysis for additional multi-parametric outputs was done using a protocol created in the MetaXpress custom module editor (CME). The custom module analysis first identified the spheroid using Hoechst staining. Spheroids were identified using the Find Spherical Objects function. Spheroid object sizes were characterized and compared by volume, diameter, and fluorescence intensities. Live cells were then identified by the presence of calcein AM signal or EthD-1 exclusion, and dead cells were identified by the presence of EthD-1 signal. Then, objects were connected in 3D volume by using Connect by Best Match function.

Measurements from individual cells included nuclear or cellular number, nuclear or cell volume, diameter, average intensities for calcein AM, EthD-1, or Hoechst; counts of all nuclei; and evaluation of average nuclear size, intensity, and distances between nuclei. Calcein AM- or EthD-1-positive or -negative cells were counted, and their volumes and intensities values were measured. In some cases, images were smoothed with a Gaussian filter or sharpened with Top Hat function. EC_50_ values were determined using a four-parameter curve fit from SoftMax^®^ Pro 6 software (Molecular Devices).

## Results

### Development and Optimization of the Live Cell High-Content Assay with 3D Spheroid Cultures

**Fig. 1.**
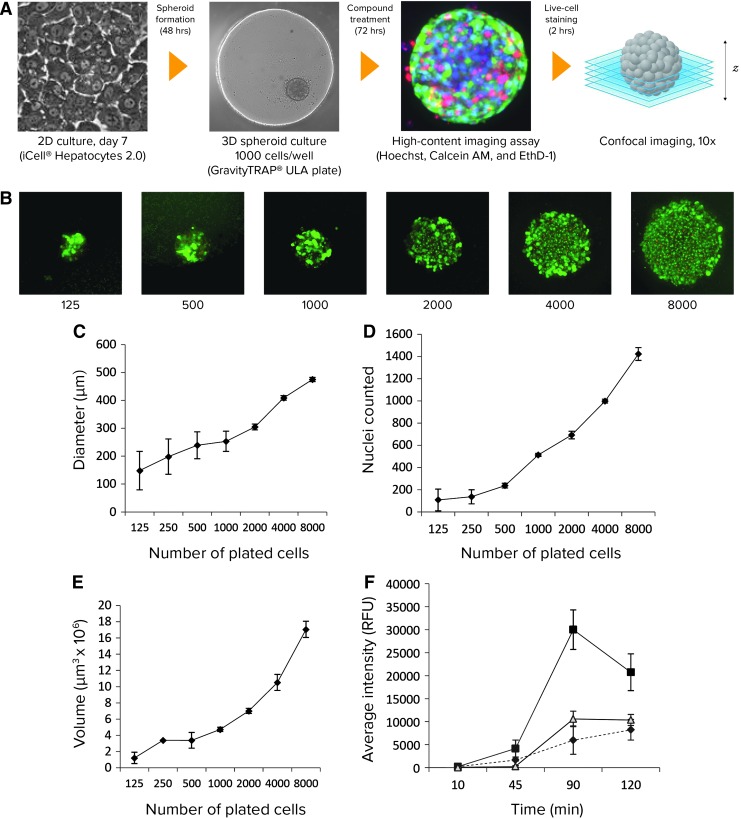
**(A)** Schematic diagram of 3D liver spheroid formation and high-content imaging assay workflow. The spheroid images shown here were from seeding with ∼1,000 cells/well. **(B)** Images of spheroids (calcein AM staining) shown for different numbers of cells plated per well (125–8,000 cells). **(C, E)** Dependence of spheroid diameter and volume on the number of cells plated (*n* = 6). **(D)** Nuclei counts of stained spheroids relative to cell plating numbers. **(F)** Average fluorescence intensities from spheroids stained with Hoechst, calcein AM, and EthD-1 for indicated times (*n* = 3). Optical filters used were DAPI (Hoechst, *solid gray*), FITC (calcein AM, *solid black*), and TexasRed (EthD-1, *dashed line*). 3D, three-dimensional.

The goal of this study was to develop and evaluate fast, accurate, and reproducible high-content imaging methods to investigate the effects of toxic compounds on the morphology and viability of 3D liver spheroids using techniques for confocal imaging and 3D image analysis. The workflow of the assay is presented in *Figure 1A*. Human iPSC-derived hepatocytes were cultured for 7 days in a 2D format before detaching the cells, supplementing the cell suspension with extracellular matrix, and plating the mixture into ULA plates to initiate 3D spheroid formation.

Both conical-shaped 96-well GravityTRAP ULA plates (InSphero) and U-shaped ULA plates (Corning) were tested for spheroid formation, compound addition, fluorescent staining, and confocal imaging. Both plates eliminate spheroid transfer steps and center the spheroids in a small focus area, facilitating capture of an entire spheroid in one 10× image. Cells aggregated at the bottom of the wells, forming spheroids within 24–48 h. The results reported in this study for iPSC-derived hepatocyte spheroids were all obtained using the GravityTRAP plates.

We studied the reproducibility of spheroid formation from iPSC-derived hepatocytes and the dependence of the spheroid size on the number of plated cells. Cells were plated at different densities (60–8,000 cells/well), incubated for 48 h, and imaged. A single spheroid per well was typically formed at each cell plating density from ∼125–8,000 cells/well, with diameter and volume sizes increasing proportionally to the number of cells plated (*Fig. 1B, C, E*).

We found that a plating density of 1,000 cells/well resulted in a consistent spheroid size and shape, with object sizes suitable for image acquisition and analysis. At this density, the average spheroid diameter after 2 days was consistent as measured by transmitted light or fluorescent imaging with a value of 220 ± 24 μm (*n* = 6), yielding a coefficient of variation (CV) of 10.9%. Smaller spheroids exhibited greater size variability (CV = 20% for 500 cells plated, *n* = 6), whereas variability in size decreased for larger spheroids (CV = 4% for 2,000 cells plated, *n* = 6). Since iPSC-derived hepatocyte spheroids do not proliferate, the diameter of untreated spheroids remained consistent in culture through the experiment.

**Fig. 2.**
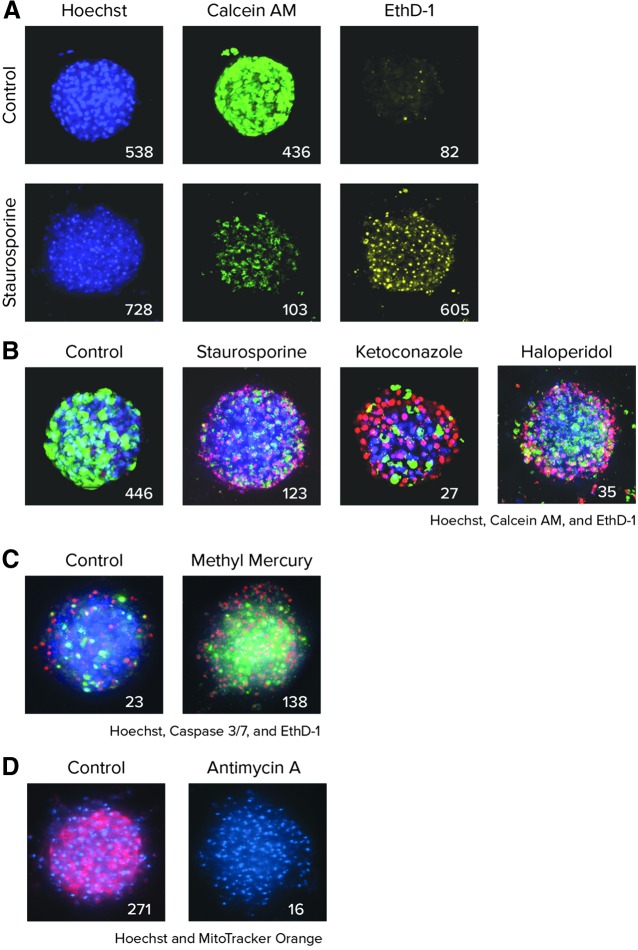
**(A)** Untreated and staurosporine-treated spheroids (1 μM) were stained with a combination of three dyes: Hoechst (10 μM), EthD-1 (3 μM), and Calcein AM (1 μM). *Numbers* represent the appropriate objects positive for each marker counted in 3D space. **(B)** Composite images of all three channels shown for the control and select compound-treated spheroids. The spheroids were treated with staurosporine (1 μM), ketoconazole (30 μM), or haloperidol (30 μM) for 72 h and images were generated using maximum projection from a Z-stack of 11 images taken at 10 μm intervals. Counted numbers of calcein AM-positive (live) cells are shown for the different treatments. **(C)** Apoptosis is apparent in the composite images of spheroids stained with CellEvent Caspase 3/7 reagent to detect caspase 3/7 (*green*), EthD-1 (*red*) for dead cells, and Hoechst (*blue*) for all nuclei. Spheroids treated for 24 h with methyl mercury (1 μM) exhibited a higher number of apoptotic cells (*green*) than the control (number of apoptotic cells is indicated). **(D)** Composite images for the control and Antimycin A (3 μM) treated spheroids stained with MitoTracker Orange (*red*) and Hoechst (*blue*). Antimycin A disrupts mitochondrial membrane integrity. Number of cells with intact mitochondria is indicated.

We had previously described staining protocols for spheroids made from transformed cell lines.^39^ Here, we used a previously developed staining protocol and concentrations with a one-step dye mixture addition that eliminates the need for fixing cells or repeated wash steps.^22,39^ Images of representative spheroids stained with a mixture of three dyes are shown in *Figure 2A, B*. Calcein AM was used to measure cell metabolic activity, viability, and a variety of morphological parameters. Hoechst was utilized to measure total cell count and nuclear shape. EthD-1 selectively penetrates cells with damaged outer membranes and was used to mark dead or necrotic cells.

Spheroids of varying sizes were tested using this protocol to confirm its suitability for 3D analysis (*Fig. 1B–E*). The raw nuclei counts relative to the cell plating densities were less than expected due to imaging limitations described next. For spheroids containing ∼1,000 cells/well (∼220 μm in diameter), the counted number of nuclei was ∼500 (*Fig. 1D*).

An additional experiment (*Fig. 1F*) demonstrated the time dependency of dye penetration into spheroids, with staining times of 90–120 min required to achieve maximal signal intensity. Spheroid imaging could be accomplished for up to 6 h post-staining if plates were kept in the incubator (data not shown). Additional staining protocols were evaluated for apoptosis (Caspase 3/7, *Fig. 2C*) and mitochondria integrity (MitoTracker Orange, *Fig. 2D*) dyes.

### Imaging 3D Spheroids

A challenge in performing spheroid assays is the ability to image and analyze cells in 3D. Common issues previously described include poor light penetration into or out of the spheroid center, light scattering by cells, and high background from out-of-plane fluorescence.^40^ These issues tend to be less important for smaller spheroids (∼220 μm) used in the present study.

Confocal microscopy provides efficient background rejection and sharper images. In this study, images were taken using an automated confocal microscope with a large field-of-view camera. Imaging protocols were additionally optimized for 3D image analysis. The microscopy system effectively captures spheroids in a single image using a 10× objective. At 10× magnification, we captured either 11 images with 10 μm distance or 17 images with 5 μm distance, which resulted in comparable cell counts. Images acquired with a 20× objective allowed better visualization of details, but they did not provide a significant difference in cell count.

Because of the limitation of sufficient light penetration into dense objects, the approximate depth of imaging into the hepatocyte spheroids prepared and stained as described earlier was ∼110 microns. Although longer staining times up to about 2 h improved dye penetration and average signal intensities (*Fig. 1F*), no further increase in light penetration or depth of view was obtained. Therefore, for spheroids containing ∼1,000 cells/well (∼220 μm diameter), visualization and enumeration of objects (cells, as defined) within the object yields approximately half of the actual number of cells present in the intact spheroid (∼500 counted). This can be illustrated by *Figure 1D*, showing dependence of the number of cells counted in spheroids from the number of cells plated initially.

In contrast, for bigger spheroids, total cell number is undercounted due to the limitations of the light penetration into samples. In the case of disintegrated, deformed, or “loosened up” spheroids, the same depth of penetration might produce an artificially higher cell count based on analysis of more than a half of the compromised spheroid. The MaxPro algorithm, which combines the highest intensity pixels along lines orthogonal to the image planes into a single image, was used for cell count comparisons. As expected, nuclei counted in 2D MaxPro represented only a fraction of nuclei counted by 3D analysis (data not shown).

### 3D Analysis of Spheroid Images

**Fig. 3.**
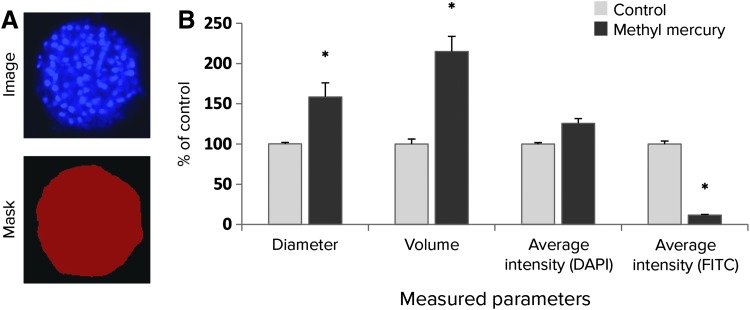
**(A)** Analysis functions segment objects in 3D volume using predetermined size and intensity thresholds. **(B)** Analysis of the spheroid as a single object. Phenotypic measurements can then be calculated, including: spheroid diameter, spheroid volume, and average intensities for nuclear (DAPI channel) and viability staining (FITC channel). Here, values were determined for the control (*light gray*) and methyl mercury treated (1 μM, *dark gray*) spheroid wells. The values were normalized to the corresponding control values that were set at 100%. Error bars represent standard deviations (*n* = 3). Values significantly different from controls (*P < 0.05, T-test) are marked with asterisks.

In the present study, 3D analysis of images was accomplished using recently added features of MetaXpress software. The software converts a stack of 2D images obtained by confocal acquisition into 3D space with appropriate detection and segmentation of objects. Spherical object analysis allowed for measuring several parameters that are important for phenotypic characterization of drug-induced hepatotoxicity, including spheroid diameter size, volume, and live/dead cell fluorescence marker intensities (*Fig. 3*). For example, spheroids treated with methyl mercury were readily distinguished from the vehicle control by significant morphological changes in spheroid size and volume (*i.e.*, toxicity-induced loss of spheroid integrity leading to gross enlargement of the 3D object) as well as by decreased viability staining (FITC channel intensity).

**Fig. 4.**
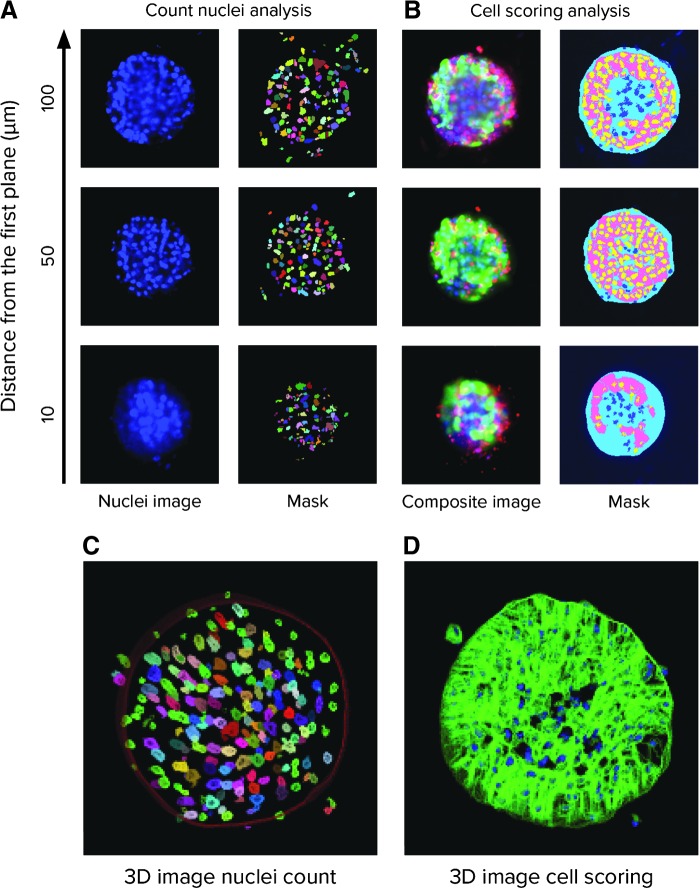
**(A)** Confocal images of a spheroid stained with Hoechst acquired using a 10 × Plan Fluor objective as a Z-stack of 11 images 10 μm apart, with three selected planes shown. Results from image analysis object masks are shown to the right of each image, with individual nuclei segmentation given a random pseudo-color. **(B)** Confocal images of the same spheroids showing the overlays of Hoechst (*blue*), calcein AM (*green*), and EthD-1 (*red*) staining. The resulting object masks are shown to the *right* of each image, with masks for the entire spheroid *light blue*, viable cell nuclei indicated in *yellow*, dead cell nuclei in *dark blue*, and cytoplasm of viable cells shown in *pink*. **(C)** Examples of 3D masks for nuclei and **(D)** calcein AM-positive cytoplasm.

The 3D analysis allows for defining individual nuclei and cells as well as for appropriate characterization of the individual cells and subcellular objects. *Figure 4* illustrates how more detailed segmentation was performed, for example, to identify and count nuclei within a spheroid and score the results for live/dead populations based on the staining patterns obtained for each marker dye. Briefly, individual Z-planes were first segmented and analyzed as 2D images, for example, for nuclei count, live/dead, or cell scoring features; then, the objects were “Connected by Best Match” function with user-defined maximum displacement of each object relative to another one (*e.g.*, 5–10 microns for the maximum displacement of the nuclei and 20–30 microns for cytoplasm). Nuclei or individual cells were, thus, segmented and scored in the context of a layered 3D volume, ideally without missing objects or counting the same objects twice.

These steps allow defining and counting the number of total cells, number of calcein AM-positive or -negative cells, and number of ethidium homodimer-positive and -negative cells. In addition, the intensities of individual cells can be defined in different colors, as well as cell volumes, diameters, distances between objects, or spatial locations of objects in 3D matrix. Significantly, the larger spheroid mask can be used to define the numbers of smaller objects within the spheroid, or externally.

### Phenotypic Analysis of Compound Treatment Effects

**Fig. 5.**
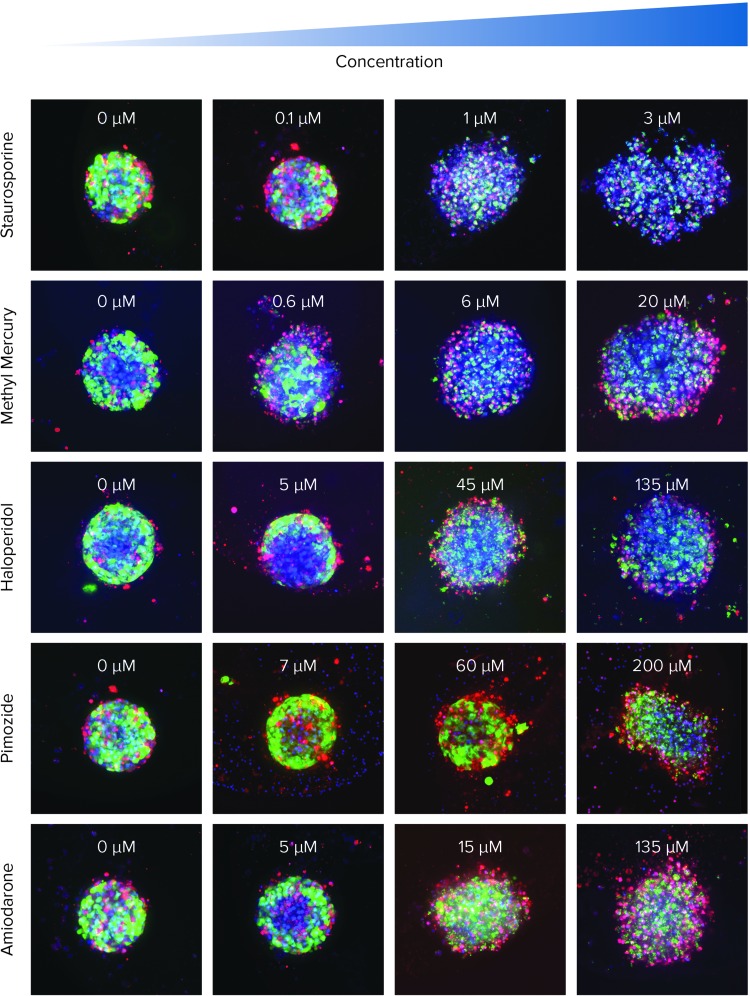
Representative images of spheroids seeded at 1,000 cells/well and treated with different compounds for 72 h (concentrations indicated). Composite images of Hoechst (*blue*), calcein AM (*green*), and EthD-1 (*red*) are presented. Quantitation is presented in *Figure 6*.

Significant changes in spheroid phenotypes and cell content were observed after compound treatment. Many spheroids lost their spherical shape, appeared disintegrated, “loose,” “flattened,” or “irregular,” had cells detached from the main body, or exhibited condensed nuclei due to cell death. These phenotypic changes in the spheroids after compound treatment occurred over a range of concentrations (*Fig. 5*).

**Fig. 6.**
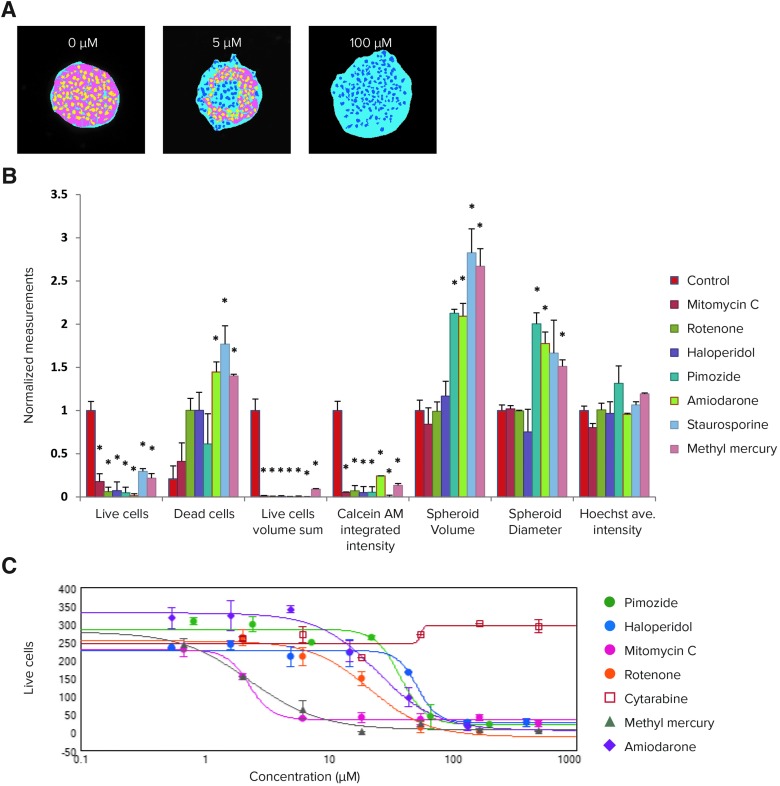
**(A)** Image analysis masks for a single plane shown for amiodarone-treated (5 and 100 μM) and control spheroids. Spheroid masks are indicated in *blue*, live cell nuclei are marked in *yellow*, live cell cytoplasm is shown in *pink*, and dead cell nuclei are marked *dark blue*. **(B)** Image analysis readouts were derived as a result of a cell scoring analysis performed in 3D space. Measurements: number of live cells and dead cells per spheroid, sum of the volumes of live cells (determined by calcein AM-positive staining of cytoplasm), integrated intensity for calcein AM signal, volume of spheroid, diameter of spheroid, and average intensity of the Hoechst nuclear stain. Data shown correspond to the following compounds (72 h exposure): control (0.1% dimethyl sulfoxide), mitomycin C (5 μM), rotenone (60 μM), haloperidol (45 μM), pimozide (60 μM), amiodarone (45 μM), staurosporine (1 μM), and methyl mercury (20 μM). All values are presented as normalized relative to the control, with the exception of the number of dead cells that was normalized to the number of total counted cells in the control. Error bars represent the standard deviations of the replicates (*n* = 3). Significant differences from control (*P* < 0.05) are indicated on the graph with asterisks. Representative images for the corresponding treatments can be found in *Figure 5*. **(C)** The dose–dependent effects and the four-parameter curve fits for the selected compounds for the 3D spheroid toxicity assay using the number of live cells (calcein AM-positive cells) per spheroid. The number of live cells was determined by the Cell Scoring analysis of images using the 3D analysis features (*n* = 3). IC_50_ values are included in *Table 1*.

For example, for amiodarone-treated spheroids, there was a notable increase in spheroid volume and diameter, as well as a decrease in the intensity of the live-cell calcein AM signal. Quantitative analysis of the images included derivation of parameters to assess morphological features of spheroids, cell content, and complexity (*Fig. 6A*). The spheroid volume, diameter, and fluorescence intensities were measured, along with the number of calcein AM-positive “live” cells, the number of EthD-1-positive “dead” cells, as well as the average volumes and intensities of cells exhibiting these markers (*Fig. 6B*).

Total cell number did not tend to decrease in response to compound effects. Instead, a concentration-dependent decrease in the number of viable cells was observed concomitant with an increase in the number of dead cells. Average fluorescent intensity for calcein AM was dramatically reduced for the entire spheroid as well as for individual segmented cells (cytoplasm). In addition, the average volume of cytoplasm stained with live cell dye calcein AM was decreased (live cell volume), as well as the sum of the volumes of calcein AM-positive cells.

For certain compound treatments such as methyl mercury and staurosporine, there was an increase in Hoechst fluorescence intensity, likely indicating nuclear condensation and apoptosis. An increase in the distance between the nuclei was also observed, and this could be attributed to a loss of cell-to-cell junctions. When using calcein AM, the number of metabolically active live cells, the sum of the volumes of the live cells, or their integrated fluorescent intensities provided the greatest sensitivity for assessment of cytotoxicity, as indicated by the largest fold changes between the control and treated samples (*Fig. 6B*).

MetaXpress CME was employed for multi-parametric image analysis. Spheroid diameter, spheroid volume, or count of dead cells changed significantly for some compounds, but typically did not follow four-parametric concentration-dependent responses. In contrast, the live cell number, the sum of their volumes, and the calcein AM integrated fluorescence intensities resulted in the largest assay windows, statistically significant differences between treated and control samples, and successfully fit into four-parameter dose–response curve models. Z′-factors, indicators of data quality, for the different readouts are presented in *Supplementary Table S2*.

For an investigation into the cytotoxicity mechanisms, we also evaluated the apoptotic phenotype and mitochondria integrity. Activation of apoptosis using Caspase 3/7 dye was measured 24 h after compound treatment. Typical activated caspase 3/7 staining patterns for control cells, and those treated with methyl mercury, are shown in *Figure 2C*. Treatment with compounds causing apoptosis resulted in an increase of caspase 3/7 staining intensity and the number of caspase 3/7 positive (apoptotic) cells. Mitochondrial potential was evaluated with MitoTracker Orange dye. Treatment of spheroids with compounds affecting mitochondria integrity resulted in a decrease of the intensity of MitoTracker Orange staining and a decrease of cells with intact mitochondria (*Fig. 2D*).

### Effects of Known Hepatotoxic Compounds

Spheroid assay performance was characterized using a representative set of 48 compounds that included known cytotoxic and hepatotoxic agents, plus six chemicals considered “safe” or nontoxic. *Table 1* presents IC_50_ values for different classes of hepatotoxic compounds: known liver toxins (aflatoxin, rotenone, methyl mercury), anti-cancer drugs (*e.g.*, doxorubicin, mitomycin), anti-depressants, anti-psychotics (*e.g.*, paroxetine, fluoxetine, thioridazine, haloperidol), anti-malarial agents (quinacrine, chloroquine), and several other types of drugs. Of the 42 selected known hepatotoxins in the set, 36 elicited detectable toxicity effects in the spheroid hepatotoxicity assay (86% sensitivity), whereas all compounds considered “safe” had no effect on the number of live cells or spheroid phenotype (100% predictivity).

**Table 1.** Comparison of Compound Responses Between 3D and 2D Cultures of iPSC-Derived Hepatocytes

	iCell hepatocytes		
Compounds	IC_50_ (μM)^a^ 3D assay	IC_50_ (μM) 2D assay	Compound description
Toxic substances			
TAB	1.69 ± 0.082	1.22 ± 1.02	Toxin
Methyl mercury	2.82 ± 0.49	5.05 ± 1.31	Toxin
Fuzariotoxin	0.481^b^	3.75	Toxin
Aflatoxin B1	0.595 ± 0.145	1.7	Toxin
Aflatoxin G1	7.78 ± 2.13	9.45 ± 2.01	Toxin
Rotenone	21.4 ± 6.9	n.d.^c^	Pesticide
Anti-proliferative agents			
Mitoxantrone	**0.533** ± **0.011^d^**	**3.7** ± **0.32**	Topoisomerase II inhibitor
Daunorubicin	0.671 ± 0.046	0.5	DNA-intercalator, anti-cancer
Idarubicin	1.11	0.94	DNA-intercalator, anti-cancer
Doxorubicin·HCl	5.69 ± 4.04	2.17 ± 0.08	DNA-intercalator, anti-cancer
Staurosporine	0.91 ± 0.12	1.71 ± 0.87	Kinase inhibitor, apoptosis inducer
Mitomycin C	2.28 ± 0.25	2.36 ± 0.65	DNA-intercalator, anti-cancer
Other drugs			
Quinacrine	1.029 ± 0.212	2.52	Anti-malaria
Coralgil	1.24	6.71	Inducer of steatosis
Puromycin	3.82	7.2 ± 11.2	Protein synthesis inhibitor
Amlodipine	4.12	30.2	Ca blocker
rac Perhexiline Maleate	6.36 ± 0.15	9.89	Mitochondria inhibitor
Tamoxifen	12.4	10.23	Estrogen receptor antagonist
Miconazole nitrate	13.71 ± 0.789	103.1 ± 132	Anti-fungal
Fluphenazine di·HCl	13.0 ± 0.824	29.5	Anti-psychotic
Paroxetine	14.73 ± 3.92	30.4	Antidepressant
Thioridazine	15.83 ± 4.47	31.4	Anti-psychotic
Chloroquine	18.60 ± 0.247	38.5	Anti-malaria
Ketoconazole	20.4 ± 10.7	61.0 ± 46.2	Anti-fungal
Amiodarone·HCl	22.6 ± 9.68	15.3 ± 18.3	Anti-arrhythmia, autophagy inducer
Paclitaxel	26.2 ± 6.39	No tox (100)^e^	Microtubule inhibitor
Fluoxetine	28.9	10.17 ± 0.321	Antidepressant
Imatinib	36.8	n.d.	Kinase inhibitor
Pimozide	35.1 ± 1.39	13.5 ± 15.9	Anti-psychotic
Apigenin	36.9 ± 10.2	n.d.	Autophagy inducer
Haloperidol·HCl	52.2 ± 21.6	No tox (100)	Anti-psychotic
Isradipine	168 ± 137	n.d.	Ca blocker
Colchicine	>100^f^	>100	Microtubule inhibitor
Tolcapone	>100	>100	Parkinson disease drug
Doxocycline	>100	>100	Antibiotic
Retinoic acid	∼500	480	Retinoic acid receptor agonist
Imipramine	No tox (100)	>100	Antidepressant (TCA)
Carboplatin	No tox (500)	No tox (500)	DNA repair, anti-cancer
Cytarabine	No tox (1,000)	No tox (1,000)	DNA intercalator, anti-cancer
Etoposide	No tox (1,000)	No tox (1,000)	Topoisomerase inhibitor, anti-cancer
Phenylbutazone	No tox (100)	No tox (100)	Nonsteroidal anti-inflammatory
Acetaminophen	No tox (1,000)	No tox (1,000)	Nonsteroidal anti-inflammatory
Negative controls			
Penicillin V potassium salt	No tox (1,000)	No tox (1,000)	Antibiotic
Streptomycin sulfate	No tox (1,000)	No tox (1,000)	Antibiotic
Aspirin	No tox (1,000)	No tox (1,000)	Anti-inflammatory
Ampicillin	No tox (1,000)	No tox (1,000)	Antibiotic
Sucrose	No tox (1,000)	No tox (1,000)	Carbohydrate
Sorbitol	No tox (1,000)	No tox (1,000)	Carbohydrate

^a^ IC_50_ values (in μM) measured for tested compounds using number of live cells per spheroid as the readout. Error limits are standard error of the parameter estimate defined from the curve fit.

^b^ The undefined standard errors for some parameters indicate that although the curve fits have converged, the uncertainty in the parameter estimates could not be determined.

^c^ n.d., not determined.

^d^ Difference between 3D and 2D is significant (*P* < 0.05). Indicated in bold.

^e^ “No tox” means that no apparent effects were observed at the highest concentration tested (value in μM in parentheses).

^f^ “>100” means that toxic effects (decrease of calcein AM-positive cells) were observed at the highest concentration tested (100 μM), but IC_50_ values were not determined.

2D, two-dimensional; 3D, three-dimensional; iPSC, induced pluripotent stem cell; TAB, tetraoctylammonium bromide.

We also compared the IC_50_ values obtained from 3D cultures versus those obtained from 2D cultures of iPSC-derived hepatocytes (*Table 1*), which had also been treated with compounds for 72 h. With some notable exceptions (*e.g.*, mitoxantrone, *P* < 0.05), the majority of the IC_50_ values were not statistically significantly different (*P* > 0.05, or not determined) between the 3D and 2D cultures. It is noteworthy that the standard errors were not calculated for many of the compound treatments, thus preventing a complete evaluation of differences in IC_50_ between the 3D and 2D cultures. However, 21 of the 33 compounds with detectable effects in both 3D and 2D formats yielded slightly lower IC_50_ values for the 3D cultures, suggesting a possible trend toward stronger toxicity effects in 3D culture.

We also compared effects for 23 selected compounds on spheroids made from transformed liver cells. Differences observed were related to the continuous proliferation of HepG2 cells in 3D culture. HepG2 spheroids were larger in size (300–350 μm diameter per 1,000 plated cells) in comparison with spheroids composed of iPSC-derived hepatocytes, since HepG2 cells appear to continuously grow in the culture during compound incubation. Also, there was a marked decrease in the size of HepG2 spheroids as well as a decrease in total cell number, in contrast to the nondividing iCell hepatocyte spheroids that did not typically decrease in size or cell number in response to compound treatment. A comparison of IC_50_ values for 23 compounds between the two cell types is given in *Table 2*. There were significant differences (*P* < 0.05) between the data with respect to several anti-proliferative compounds (*e.g.*, staurosporine, paclitaxel, and mitomycin). The IC_50_s determined for HepG2 spheroid cultures were in the nanomolar range, whereas the iPSC-derived hepatocyte spheroids either displayed markedly right-shifted IC_50_s or were unaffected by these types of agents. With occasional exceptions (*e.g.*, methyl mercury, pimozide, tetraoctylammonium bromide), the majority of the hepatotoxic compounds acting via mechanisms that do not inhibit replication (*e.g.*, haloperidol and ketoconazole) showed similar effects in both cell models.

**Table 2.** Comparison of Compound Responses Between 3D Cultures of HepG2- and iPSC-Derived Hepatocytes

	HepG2, IC_50_ (μM)^a^	iPSC-derived hepatocytes, IC_50_ (μM)
Anti-proliferation agents		
Idarubicin	0.00513 ± 0.00523	1.11
Staurosporine	**0.00582 ± 0.00098^b^**	**0.91 ± 0.12**
Doxorubicin·HCl	0.008 ± 0.0005	5.69 ± 4.04
Mitomycin C	**0.012 ± 0.0006**	**2.28 ± 0.25**
Paclitaxel	**0.01 ± 0.0012**	**26.2 ± 6.39**
Cytarabine	0.307 ± 0.097	No tox (1,000)^c^
Etoposide	4.18 ± 2.99	No tox (1,000)
Carboplatin	15.82 ± 2.61	No tox (1,000)
Toxic substances		
TAB	**0.005 ± 0.0047**	**1.69 ± 0.082**
Methyl mercury	**9.19 ± 2.98**	**2.82 ± 0.49**
Rotenone	8.32 ± 4.37	21.4 ± 6.94
Other drugs		
Pimozide	**4.73 ± 3.18**	**35.1 ± 1.39**
Tamoxifen	18.7 ± 29.4	12.4 ± 13.2
Ketoconazole	19.2 ± 12.5	20.4 ± 10.7
Apigenin	21.9 ± 13.4	36.9 ± 10.18
Isradipine	22.17	168 ± 137
Haloperidol·HCl	25.08 ± 5.50	52.2 ± 21.6
Chloroquine	33.28	18.60 ± 0.247
Amiodarone·HCl	42.4 ± 8.39	22.6 ± 9.68
Imatinib	178 ± 40.54	36.8
Negative controls		
Aspirin	No tox (1,000)	No tox (1,000)
Ampicillin	No tox (1,000)	No tox (1,000)
Sucrose	No tox (1,000)	No tox (1,000)

^a^ IC_50_ values (in μM) were determined for tested compounds using number of live cells per spheroid as the readout. Error limits are standard error of the parameter estimate defined from the curve fit.

^b^ Differences between HepG2- and iPSC-derived hepatocytes is significant (*P* < 0.05) for compounds indicated in bold.

^c^ “No tox” means that no apparent effects were observed at the highest concentration tested (value in μM in parentheses).

## Discussion

Human 3D tissue models derived from iPSC-differentiated cells can provide useful systems for analyzing the pathogenesis of diseases or drug-induced toxic effects.^11,41,42^ Although *in vivo* toxicity assessment remains the “gold standard,” 3D models can accelerate the process by enabling rapid compound prioritization for *in vivo* testing and establishing mechanisms of action. Although interest in 3D cell models is increasing rapidly, the currently limited characterization of 3D assays impedes widespread adoption. Relative to established 2D methods, 3D methods are less developed with respect to imaging and analysis, and compatibility with screening systems. There are still important limitations to 3D systems, including imaging and analysis, sensitivity, and compatibility with screening systems.

The aim of the present study was to develop and characterize confocal imaging and 3D analysis methods that would be compatible with automated instruments and show feasibility of using 3D spheroid cultures for toxicity screening. In contrast to previous studies, we have focused here on optimizing higher resolution imaging and 3D analysis. We have detailed the analysis protocol and characterized the cell population analysis method that allows multi-parametric quantification of different phenotypes. We have shown how 3D image analysis can deliver a number of informative phenotypic readouts that enable screening for deleterious effects of test compounds on cell morphology and viability from a simple assay workflow.

Using a set of representative hepatotoxic compounds, we demonstrated how different readouts can be used in combination to characterize complex drug-induced effects on aspects such as spheroid morphology (*e.g.*, changes in size or volume), integrity (*e.g.*, changes in the distance between cells), viability (*e.g.*, live/dead cell counts), and other parameters (*e.g.*, average fluorescence intensity) to assess toxicity effects (*Figs. 2–4*). IC_50_ values from different readouts showed that counting the number of calcein AM-positive (metabolically active) cells in spheroids provided the greatest sensitivity to compound effects and yielded the best curve fits for ranking compound toxicities.

Other parameters measured would provide supporting information about specific drug-induced phenotypes, for example, spheroid size, integrity, or content of dead cells. It should also be noted that due to better light penetration into deformed spheroids, it is possible that they might yield higher live cell counts, resulting in an underestimation of certain IC_50_ values. However, deformed spheroids are typically expected to contain few live cells that would minimize the effect of overcounting live cells on IC_50_ values.

A comparison of compound toxicity effects between iPSC-derived hepatocytes and HepG2 spheroid cultures demonstrated significant differences in assay response. Not surprisingly, the cancer-derived HepG2 cell line was >100× more sensitive to compounds with anti-proliferative effects. Thus, the rank order for toxicity was also very different, with HepG2 cells ranking the anti-proliferative compounds as more toxic. In addition, the observed phenotypic changes were also very different. A decrease in spheroid size and total cell number for HepG2 spheroids was not observed for iPSC-derived hepatocytes. These observations suggest that HepG2 spheroids represent a very different model that reflects special properties of cancer cells that are not present in primary cells.

In addition, the iPSC-derived hepatocyte 3D spheroid assay results were compared with results obtained using a typical 2D culture format. It has been previously shown that 3D cultures can often be more or less sensitive than 2D cultures to toxicity effects, and that these relative sensitivities correlate with compound mechanisms of action.^1,9,11,32^ Nevertheless, the studies here found that the IC_50_ values (*Table 1*) obtained for many of the compounds in this small test set were comparable between the 3D spheroids and the 2D culture, and the rank order of compound potency was similar.

However, since the assays here were conducted over relatively brief exposure times, it is possible that more functional differences between the 2D and 3D formats could be revealed if the treatments were extended over longer periods. It is also possible that testing a larger set of compounds, including compounds shown to elicit marginal toxicity effects in 2D, could identify additional differences between the two models. Similarly, drugs influenced by CYP450 activity or other metabolic processing events may respond differently in 3D systems owing to enhanced functions. Some comparisons of long-term drug-induced toxicities in 3D versus 2D cultures have recently been noted using primary cells (InSphero^23^), which demonstrated the importance of extended exposure times to better discriminate compound effects between 2D and 3D culture formats.

Going forward, we anticipate successful applications of the method described here to high-content assays utilizing other cell types and additional markers for readouts such as hypoxia, cytoskeleton, and kinase activation. The approach may also be extensible to more complex 3D systems, such as cultures containing multiple cell types (*e.g.*, Kuppfer cells, fibroblasts, endothelial cells) where 3D analysis would allow characterization of different cell populations and their roles in toxicity and liver injury. In summary, the 3D spheroid cell model combined with high-content 3D assays presented here shows promise as a sensitive and reproducible screening tool for assessing hepatotoxicity. Although the predictivity of the assay in comparison with animal and clinical data still needs to be established, further development of the methods and models will increase the utility of this *in vitro* tool for screening.

## Supplementary Material

Supplemental data

## Abbreviations Used

3D: three-dimensional
2D: two-dimensional
ATP: adenosine triphosphate
CME: custom module editor
CV: coefficient of variation
DAPI: 4′,6-diamidino-2-phenylindole
DMSO: dimethyl sulfoxide
EthD-1: ethidium homodimer-1
FITC: fluorescein isothiocyanate
iPSC: induced pluripotent stem cell
MaxPro: maximum projection
SD: standard deviation
TAB: tetraoctylammonium bromide
TRITC: tetramethyl rhodamine isothiocyanate
ULA: ultra-low attachment

## References

[1] Hartung T: Toxicology for the twenty-first century. Nature 2009;460:208–212 19587762 10.1038/460208a

[2] Atienzar FA, Novik EI, Gerets HH, *et al.*: Predictivity of dog co-culture model, primary human hepatocytes and HepG2 cells for the detection of hepatotoxic drugs in humans. Toxicol Appl Pharmacol 2014;275:44–61 24333257 10.1016/j.taap.2013.11.022

[3] Gerets HH, Tilmant K, Gerin B, *et al.*: Characterization of primary human hepatocytes, HepG2 cells, and HepaRG cells at the mRNA level and CYP activity in response to inducers and their predictivity for the detection of human hepatotoxins. Cell Biol Toxicol 2012;28:69–87 22258563 10.1007/s10565-011-9208-4 PMC3303072

[4] Ramsden D, Tweedie DJ, Chan TS, Taub ME, Li Y: Bridging in vitro and in vivo metabolism and transport of faldaprevir in human using a novel cocultured human hepatocyte system, HepatoPac. Drug Metab Dispos 2014;42:394–406 24366904 10.1124/dmd.113.055897

[5] Chang TT, Hughes-Fulford M: Monolayer and spheroid culture of human liver hepatocellular carcinoma cell line cells demonstrate distinct global gene expression patterns and functional phenotypes. Tissue Eng Part A 2009;15:559–567 18724832 10.1089/ten.tea.2007.0434 PMC6468949

[6] Wang W, Itaka K, Ohba S, *et al.*: 3D spheroid culture system on micropatterned substrates for improved differentiation efficiency of multipotent mesenchymal stem cells. Biomaterials 2009;30:2705–2715 19215979 10.1016/j.biomaterials.2009.01.030

[7] Wang J, Chen F, Liu L, *et al.*: Engineering EMT using 3D micro-scaffold to promote hepatic functions for drug hepatotoxicity evaluation. Biomaterials 2016;91:11–22 26994875 10.1016/j.biomaterials.2016.03.001

[8] Drewitz M, Helbling M, Fried N, *et al.*: Towards automated production and drug sensitivity testing using scaffold-free spherical tumor microtissues. Biotechnol J 2011;6:1488–1496 22102438 10.1002/biot.201100290

[9] Tung YC, Hsiao AY, Allen SG, Torisawa YS, Ho M, Takayama S: High-throughput 3D spheroid culture and drug testing using a 384 hanging drop array. Analyst 2011;136:473–478 20967331 10.1039/c0an00609b PMC7454010

[10] Al-Qubaisi M, Rozita R, Yeap SK, Omar AR, Ali AM, Alitheen NB: Selective cytotoxicity of goniothalamin against hepatoblastoma HepG2 cells. Molecules 2011;16:2944–2959 21471934 10.3390/molecules16042944 PMC6260619

[11] Kunz-Schughart LA, Freyer JP, Hofstaedter F, Ebner R: The use of 3-D cultures for high-throughput screening: the multicellular spheroid model. J Biomol Screen 2004;9:273–285 15191644 10.1177/1087057104265040

[12] Dunn JC, Yarmush ML, Koebe HG, Tompkins RG: Hepatocyte function and extracellular matrix geometry: long-term culture in a sandwich configuration. FASEB J 1989;3:174–177 2914628 10.1096/fasebj.3.2.2914628

[13] Anson BD, Kolaja KL, Kamp TJ: Opportunities for use of human iPS cells in predictive toxicology. Clin Pharmacol Ther 2011;89:754–758 21430658 10.1038/clpt.2011.9 PMC3593635

[14] Elkayam T, Amitay-Shaprut S, Dvir-Ginzberg M, Harel T, Cohen S: Enhancing the drug metabolism activities of C3A—a human hepatocyte cell line—by tissue engineering within alginate scaffolds. Tissue Eng 2006;12:1357–1368 16771648 10.1089/ten.2006.12.1357

[15] Gomez-Lechon MJ, Castell JV, Donato MT: The use of hepatocytes to investigate drug toxicity. Methods Mol Biol 2010;640:389–415 20645064 10.1007/978-1-60761-688-7_21

[16] Groneberg DA, Grosse-Siestrup C, Fischer A: In vitro models to study hepatotoxicity. Toxicol Pathol 2002;30:394–399 12051557 10.1080/01926230252929972

[17] Li L, Zhou Q, Voss TC, Quick KL, LaBarbera DV: High-throughput imaging: focusing in on drug discovery in 3D. Methods 2016;96:97–102 26608110 10.1016/j.ymeth.2015.11.013 PMC4766031

[18] Medine CN, Lucendo-Villarin B, Storck C, *et al.*: Developing high-fidelity hepatotoxicity models from pluripotent stem cells. Stem Cells Transl Med 2013;2:505–509 23757504 10.5966/sctm.2012-0138 PMC3697818

[19] O'Brien PJ, Irwin W, Diaz D, *et al.*: High concordance of drug-induced human hepatotoxicity with in vitro cytotoxicity measured in a novel cell-based model using high content screening. Arch Toxicol 2006;80:580–604 16598496 10.1007/s00204-006-0091-3

[20] Presley AD, Fuller KM, Arriaga EA: MitoTracker Green labeling of mitochondrial proteins and their subsequent analysis by capillary electrophoresis with laser-induced fluorescence detection. J Chromatogr B Analyt Technol Biomed Life Sci 2003;793:141–150 10.1016/s1570-0232(03)00371-4 12880861

[21] Scotter EL, Narayan P, Glass M, Dragunow M: High throughput quantification of mutant huntingtin aggregates. J Neurosci Methods 2008;171:174–179 18367250 10.1016/j.jneumeth.2008.02.007

[22] Sirenko O, Hesley J, Rusyn I, Cromwell EF: High-content assays for hepatotoxicity using induced pluripotent stem cell-derived cells. Assay Drug Dev Technol 2014;12:43–54 24229356 10.1089/adt.2013.520 PMC3934660

[23] Messner S, Agarkova I, Moritz W, Kelm JM: Multi-cell type human liver microtissues for hepatotoxicity testing. Arch Toxicol 2013;87:209–213 23143619 10.1007/s00204-012-0968-2 PMC3535351

[24] Berger DR, Ware BR, Davidson MD, Allsup SR, Khetani SR: Enhancing the functional maturity of induced pluripotent stem cell-derived human hepatocytes by controlled presentation of cell-cell interactions in vitro. Hepatology 2015;61:1370–1381 25421237 10.1002/hep.27621

[25] Mann DA, Einhorn S., Block K: Human iPSC-derived hepatocytes: functional model tissue for in vitro predictive metabolism, toxicity, and disease modeling. Gen Eng Biotech News 2013;33:28–29

[26] Mann DA. Human induced pluripotent stem cell-derived hepatocytes for toxicology testing. Expert Opin Drug Metab Toxicol 2015;11:1–5 25385341 10.1517/17425255.2015.981523

[27] Lu J, Einhorn S, Venkatarangan L, *et al.*: Morphological and functional characterization and assessment of iPSC-derived hepatocytes for in vitro toxicity testing. Toxicol Sci 2015;147:39–54 26092927 10.1093/toxsci/kfv117

[28] Kim JH, Jang YJ, An SY, *et al.*: Enhanced metabolizing activity of human ES cell-derived hepatocytes using a 3D culture system with repeated exposures to xenobiotics. Toxicol Sci 2015;147:190–206 26089346 10.1093/toxsci/kfv121

[29] Takebe T, Zhang RR, Koike H, *et al.*: Generation of a vascularized and functional human liver from an iPSC-derived organ bud transplant. Nat Protoc 2014;9:396–409 24457331 10.1038/nprot.2014.020

[30] Domansky K, Inman W, Serdy J, Dash A, Lim MH, Griffith LG: Perfused multiwell plate for 3D liver tissue engineering. Lab Chip 2010;10:51–58 20024050 10.1039/b913221j PMC3972823

[31] Takebe T, Sekine K, Enomura M, *et al.*: Vascularized and functional human liver from an iPSC-derived organ bud transplant. Nature 2013;499:481–484 23823721 10.1038/nature12271

[32] Vinci M, Gowan S, Boxall F, *et al.*: Advances in establishment and analysis of three-dimensional tumor spheroid-based functional assays for target validation and drug evaluation. BMC Biol 2012;10:29 22439642 10.1186/1741-7007-10-29 PMC3349530

[33] Krausz E, de Hoogt R, Gustin E, *et al.*: Translation of a tumor microenvironment mimicking 3D tumor growth co-culture assay platform to high-content screening. J Biomol Screen 2013;18:54–66 22923784 10.1177/1087057112456874

[34] Zhang M, Luo G, Zhou Y, Wang S, Zhong Z: Phenotypic screens targeting neurodegenerative diseases. J Biomol Screen 2014;19:1–16 23958650 10.1177/1087057113499777

[35] Kim JY, Fluri DA, Marchan R, *et al.*: 3D spherical microtissues and microfluidic technology for multi-tissue experiments and analysis. J Biotechnol 2015;205:24–35 25592049 10.1016/j.jbiotec.2015.01.003

[36] Horman SR, To J, Orth AP: An HTS-compatible 3D colony formation assay to identify tumor-specific chemotherapeutics. J Biomol Screen 2013;18:1298–1308 23918920 10.1177/1087057113499405

[37] Wenzel C, Riefke B, Grundemann S, *et al.*: 3D high-content screening for the identification of compounds that target cells in dormant tumor spheroid regions. Exp Cell Res 2014;323:131–143 24480576 10.1016/j.yexcr.2014.01.017

[38] Waite CL, Roth CM: PAMAM-RGD conjugates enhance siRNA delivery through a multicellular spheroid model of malignant glioma. Bioconjug Chem 2009;20:1908–1916 19775120 10.1021/bc900228m PMC3047462

[39] Sirenko O, Mitlo T, Hesley J, Luke S, Owens W, Cromwell EF: High-content assays for characterizing the viability and morphology of 3D cancer spheroid cultures. Assay Drug Dev Technol 2015;13:402–414 26317884 10.1089/adt.2015.655 PMC4556086

[40] Hama H, Kurokawa H, Kawano H, *et al.*: Scale: a chemical approach for fluorescence imaging and reconstruction of transparent mouse brain. Nat Neurosci 2011;14:1481–1488 21878933 10.1038/nn.2928

[41] Khademhosseini A, Langer R, Borenstein J, Vacanti JP: Microscale technologies for tissue engineering and biology. Proc Natl Acad Sci U S A 2006;103:2480–2487 16477028 10.1073/pnas.0507681102 PMC1413775

[42] Langer R, Tirrell DA: Designing materials for biology and medicine. Nature 2004;428:487–492 15057821 10.1038/nature02388

