# Supplementary material for: Phenotypic Characterization of Toxic Compound Effects on Liver Spheroids Derived from iPSC Using Confocal Imaging and Three-Dimensional Image Analysis
Source: Assay Drug Dev Technol. 2016 Sep 1;14(7):381–94. doi: 10.1089/adt.2016.729 (PMC5003004; doi:10.1089/adt.2016.729)
Supplement: Supplemental data [file Supp_Data.pdf]

SUPPLEMENTARY DATA

3D IMAGE ANALYSIS

Automatic segmentation of objects in three-dimensional (3D) microscopy images is essential for many biological studies, including high-throughput analysis of morphology and phenotypes at single-cell level. The complexity and variability of the microscopy images present many difficulties to the traditional image segmentation methods. Complications also arise when nuclei are juxtaposed or connected to one another, increasing the rate of over-segmentation or under-segmentation.

Threshold, watershed, deformable models, and graph-based formulations are the basis for the most commonly used segmentation techniques for microscopic images. Straightforward approaches such as the threshold method yield poor segmentation results due to the relatively low signal-to-noise ratio and the densely packed objects. More sophisticated algorithms such as watershed, level sets, or graph-based cut produce reasonable results, but they are not feasible for high throughput 3D analysis due to the complexity that accompanies high demands on computational resources.

In this article, we used a new algorithm that is specifically designed to perform a fast parallelized extraction of blob-type objects from the raw 3D microscopy images. We approach the segmentation problem by first doing an adaptive threshold to produce coarse object segmentation. The geometrical information of the coarse segmented objects is then obtained by the 3D distance map. The 3D distance map is calculated in accordance with a method described in Felzenwalb *et al.*, “Distance Transforms of Sampled Functions,” Theory of Computing (Volume 8, 2012). The article provides a linear-time algorithm for solving minimization problems involving a cost function with both local and spatial terms.

In our implementation, we further improve the algorithm in the three following aspects: (1) In the first dimension grid, the distance to the nearest edge pixels is calculated by a fast two-pass (forward and backward) propagation under L1 norm. (2) The algorithm is modified so that each dimension can have a different resolution, which is very important for 3D microscope images, because the depth of a voxel (along the z-dimension or optical axis) has typically less than half the resolution of the pixel in the x- and y-dimension. Factors affecting axial resolution are the objective numerical aperture and pinhole diameter (for confocal system). (3) The algorithm is modified so that the voronoi map is computed in the same loop while the distance transform is calculated. The seeds identification step selects local maxi from the distance map.

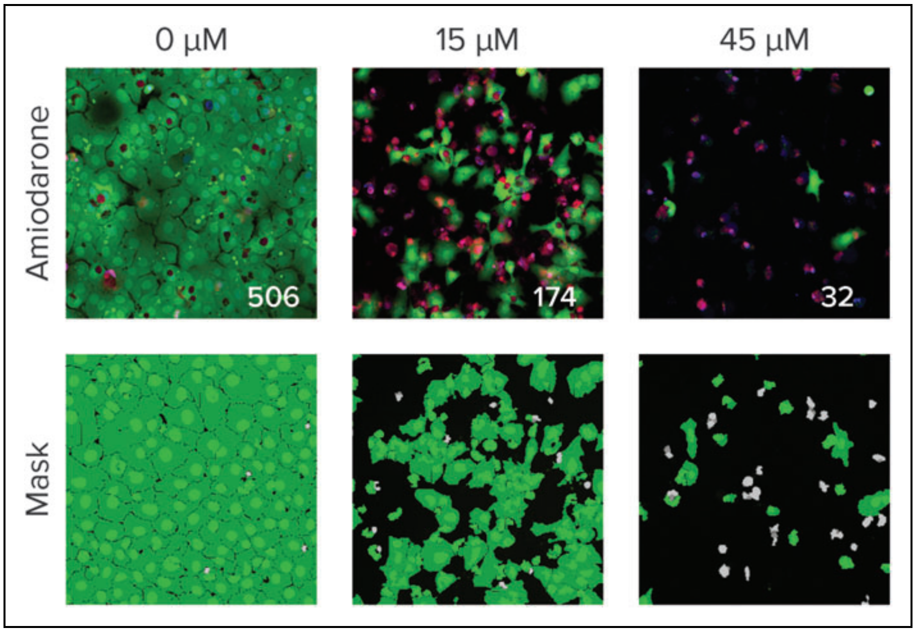

Supplementary Fig. S1. Conventional (2D) culture of iCell Hepatocytes was performed according to the protocols from Cellular Dynamics International. Cells were plated onto collagen I coated 384-well plates (10,000 cells/well) and incubated for 48 h. Next, cells were treated with compounds for 72 h. Then, cells were stained with a combination of three dyes, calcein AM, Hoechst, and EthD-1 (same concentrations that were used for 3D culture). Images were taken using 10× or 20× magnifications, wide field system, and analyzed using the Multi-wavelength Cell Scoring analysis module. Composite images of the control and amiodarone-treated cells presented in the top panel: calcein AM (green), nuclei (blue), dead nuclei (red), 20×.

Supplementary Table S1. Image Acquisition Parameters for Various Dyes					
		Bandpass (nm)		Exposure time (ms)	
Dye	Filter cube	Excitation	Emission	Widefield	Confocal
Hoechst	DAPI	377/50	447/60	20	500
Calcein AM	FITC	482/35	536/40	2	20
EthD-1	Texas Red	562/40	624/40	30	500
Caspase 3/7	FITC	482/35	536/40	20	200
MitoTracker Orange	TRITC	543/22	593/40	20	200

Supplementary Table S2. Z′-Values[a] Calculated for the Values Presented in *Figure 6B*

	Mitomycin C	Rotenone	Haloperidol	Pimozide	Amiodarone	Staurosporine	Methyl mercury
Live cells (calcein AM positive)	0.51	0.50	0.41	0.51	0.62	0.44	0.40
Dead cells (EthD-1 positive)	n.d.	n.d.	n.d.	n.d.	0.37	0.32	0.59
Positive cell volume ave	0.13	0.26	0.11	0.19	0.24	0.25	0.13
Positive cell volume sum	0.53	0.52	0.52	0.54	0.55	0.55	0.48
FITC-integrated intensity	0.16	0.35	0.14	0.16	0.21	0.40	0.23

[a]Z′-factors calculated as 1–3*(STDEVtreated+STDEVcontrol)/(AVEcontrol-AVEtreated) n = 3.
n.d., not determined.

Additional filters are applied to the local peaks to avoid over-segmentation inherent with object separation via using all the peaks as seeds. The filters applied include: (1) Minimum peak intensity: Only the peak pixels with an intensity value above threshold are under consideration. They prevent small maxima from starting new regions and help prevent over-segmentation. (2) Distance between peak pixels relative to the distance to the closest edge. Each peak pixel that is not adjacent to another peak pixel or within a predetermined Euclidean distance from one another peak pixel is classified as a marker pixel.

Object splitting step segments connected objects as identifying boundaries between regions given "seeds" in individual coarse objects. It comprises a plurality of processors operating in parallel that assigns each pixel in the coarse segmented objects to an individual seed or background according to their scores. The metric score is defined as $score_{ijk} = r - d_{ijk}$, the vector of pixel (i, j, k) to the respective seed position $x(x1, x2, x3)$ is calculated as $d_{ijk} = (x1 - i, x2 - j, x3 - k)^T$, and the r is the object radius estimated from the distance map.

Most objects are correctly segmented using the steps mentioned earlier. However, some segmented regions with an irregular shape might need to be merged or removed. The postprocessing steps use statistical and geometry connectivity information of the segmented object as the refined knowledge to correct the segmentation error.
